# Nonmotor Symptoms in Parkinson's Disease in 2012: Relevant Clinical Aspects

**DOI:** 10.1155/2012/198316

**Published:** 2012-07-25

**Authors:** Anne Marie Bonnet, Marie France Jutras, Virginie Czernecki, Jean Christophe Corvol, Marie Vidailhet

**Affiliations:** ^1^APHP, Department of Neurology, Hospital Pitié-Salpêtrière, Paris, France; ^2^INSERM, APHP, ICM, Centre d'Investigation Clinique, CIC-9503, Hospital Pitié-Salpêtrière, Paris, France; ^3^INSERM, CR-ICM, UMRS 975, Hospital Pitié-Salpêtrière, Paris, France

## Abstract

Nonmotor symptoms (NMSs) of Parkinson's disease (PD) are common, but they are often underrecognized in clinical practice, because of the lack of spontaneous complaints by the patients, and partly because of the absence of systematic questioning by the consulting physician. However, valid specific instruments for identification and assessment of these symptoms are available in 2012. The administration of the self-completed screening tool, NMSQuest, associated with questioning during the consultation, improves the diagnosis of NMSs. NMSs play a large role in degradation of quality of life. More relevant NMSs are described in this review, mood disorders, impulse control disorders, cognitive deficits, hallucinations, pain, sleep disorders, and dysautonomia.

## 1. Introduction

Nonmotor symptoms (NMS) of Parkinson's disease (PD) are common, but yet are often underrecognized in clinical practice because of the lack of spontaneous complaints by the patients, and also the absence of systematic questioning by health care professionals.

NMSs in PD have been systematically described for the first time in 2006 by Chaudhuri et al. [1]. NMS are found in a large proportion of patients with PD [2], and these symptoms consist of autonomic dysfunction, sensory complaints, neuropsychiatric disturbances, sleep disorders, fatigue, and many others (Table 1) [3].

NMS occur throughout the course of the disease. Some of them, such as depression, fatigue, and olfactory disorders, may appear at the earliest stage of the disease, in not treated patients [4]. Sometimes, NMS can even precede the motor symptoms or signs by several years and then herald the onset of PD. These premotor symptoms include olfactory dysfunction, REM sleep behaviour disorder (RBD), constipation, depression, and pain [1, 5]. On the other hand, at advanced stage of the disease, NMS coexist for most patients with motor fluctuations [6].

The frequency of NMS increases along with the disease duration [7]. At the time of diagnosis, the prevalence of NMS among PD patients is 21% (pain, urinary symptoms, depression, and anxiety) [8] and goes up to 88% after 7 years of disease progression [9]. In a recent international study, NMS such as constipation, bladder dysfunction, and feeling of sadness are reported by more than one-half of the patients, significantly more prevalent among PD patients than controls, and correlated with the duration of the disease [1, 10].

Most of the nonmotor features associated with PD are presumably related to the involvement of the nondopaminergic systems, with the hypothesis of involvement of other neurotransmitters including serotoninergic, noradrenergic, and cholinergic transmission [11]. This is the case of depression in PD, which could depend on loss of dopaminergic and noradrenergic transmission in the limbic system [12]. Indeed, nondopaminergic therapies have an important role in the treatment of most NMS.

Nevertheless, dopaminergic dysfunction involvement may contribute to many NMS [13]. Depression, anxiety, and apathy seem to be partly related to hypodopaminergic transmission [14] and are improved by levodopa therapy. On the other hand, hyperdopaminergic transmission related to dopaminergic therapy can induce NMS, like dopamine dysregulation syndrome, drug-induced hallucinations and “psychosis,” hypomania, addiction to treatment, and impulse control disorders (ICD) [15].

Nonmotor fluctuations (NMFs) are also dopamine-dependant NMS and may be improved by levodopa therapy, applying the continuous dopaminergic stimulation theory, in order to eliminate “off” periods [13]. 

Many NMS, such as depression, can result in a major disability and seriously impact the quality of life [16]. In a study of several hundred patients, the score of NMS, evaluated with the non motor symptoms scale (NMSS) [17], is correlated with the score of PDQ-39 [18]. The more severe are the NMS, the more affected is the quality of life.

These facts highlight the importance that adequate tools to detect NMS, in order to optimize the treatment of PD patients. Accordingly, a more recent review has described the instruments available for the detection and assessment of NMS, as well as recommendations for their treatment [4]. Fortunately, specific and valid instruments may help clinicians to assess these symptoms although therapeutic strategies remain limited in 2012 [19]. This paper was recently published with the objective to update previous Evidence-Based Medicine reviews on treatments for NMS in PD. Only four treatments are considered efficacious: Pramipexole for the treatment of depressive symptoms, Clozapine for the treatment of psychosis, Rivastigmine for the treatment of dementia, and botulinum toxin for the treatment of sialorrhea [19]. 

## 2. Description of Different NMS

### 2.1. Mood Disturbances and Apathy

Like any other chronic incurable conditions, PD can be discouraging for the patient and his family, but the pathology of PD in itself might predispose to anxiety and depression, through dysfunction of specific pathways. Depression, anxiety, and fatigue are not recognized by neurologists in almost one-half of consultations. Mood or behavioral disorders should be assessed by systematic questioning during the consultation and with specific scales in research context. 

#### 2.1.1. Depression

Depression occurs at any stage of the disease, even at the beginning or sometimes many years before the onset of the disease [20]. Depression can occur in up to 27.6% of PD patients during early stages of the disease [21]. There is no correlation between depression and motor disability or cognitive decline [22].

Depression may consist in major depressive disorder (17%), minor depressive disorder (22%), and dysthymia (13%), and clinical significant depressive symptoms are present in 35% of PD patients [23, 24]. However, the presentation of depression in PD is different from the classical features. In PD patients, predominant features are somatic (lack of energy, psychomotor slowing), with irritability, but no guilty or failing feeling. Depression is associated with sleep disorders, lack of refreshing sleep, decrease of libido, and feeling of poor physical appearance [22].

PD patient with depression encounters difficulties to express their emotions and considers first the motor disability. Our perception of the PD patient's disability caused by the depression has to take into account resigned complaints of patients, and their difficulties to enter into a relationship with others. Also, other PD associated symptoms may obscure the diagnosis of depression, particularly motor symptoms such as akinesia, but also apathy, and anxiety [23]. 

Depression may involve specific neuronal pathways: raphe nuclei (serotoninergic), locus coeruleus (noradrenergic), amygdala, cingular cortex, and mesolimbic and mesocortical mesothalamic pathways (dopaminergic) [20].

Antidepressant medications employed in PD depression include tricyclic antidepressants, selective serotonin reuptake inhibitors [25, 26], and therapies acting on several neurotransmitters (e.g., noradrenergic and serotoninergic medication). Depression associated with “off” periods is improved by optimization of levodopatherapy. Cognitive behavioral therapy is effective in PD depression [27].

#### 2.1.2. Anxiety

Generalized anxiety (with a feeling of situational insecurity) appears to be frequent in PD, as well as single phobia, social phobia, and panic trouble. Anxiety is two times more frequent in PD compared with the general population. The presence of anxiety symptoms has been found in 20 to 46% of PD patients [28]. During the course of the disease, 30 to 50% of PD patients experience anxiety [29, 30], which can be partly explained by the burden of the disease.

Risk factors for anxiety disorders are female gender, presence of motor fluctuations, and previous history of anxiety disorders [31]. It also occurs more frequently in young patients. Anxiety can be disabling, leading to social isolation and aggressiveness. When anxiety is present, depression is not so far [32]. Moreover, anxiety can worsen other parkinsonian symptoms, such as cognitive, but also motor symptoms.

Anxiety is often related to motor fluctuations, occurring just before intrusion of the “off” period, or being sustained during the “off” period [6].

Sometimes, anxiety can be quite challenging to diagnose, especially when manifesting as isolated aggressiveness, avoiding behaviors, expression difficulties, or cognitive disturbances.

Anxiety is often present long time before the onset of the disease, in the form of constant anxiety, panics attacks, or phobia [33].

Classically treated with benzodiazepines, anxiety is now treated with long lasting therapies, particularly serotoninergic medications also used for treatment of depression [34]. Compared to benzodiazepines, serotoninergic medication allows a long-term therapy, without inducing dependence. Anxiety linked to motor fluctuations is improved by adaptation of levodopatherapy, which reduces motor fluctuations. Adjunctive therapies such as psychotherapy are also performed. 

#### 2.1.3. Apathy

Recent literature has been focused on apathy. Apathy consists in a loss of motivation, which appears in emotional, intellectual domains and in the behavior. For the diagnosis of apathy, the decrease of spontaneous acting must not be imputable to motor disability, nor to severe cognitive decline [35, 36]. Indeed, this neuropsychiatric symptom is frequent, with a prevalence of 30% to 40% in PD patients [37, 38]. Apathy is one of the major determinants of a reduced quality of life in PD [39], even at early stages. As such, early diagnosis and efficient therapy are important in order to avoid further consequences on quality of life and disability.

In recent years, a new approach has been initiated in apathy. Apathy can be assessed by the decrease of voluntary behaviors [40], that is, behaviors effectively decided by the patient himself, and not induced by surrounding people. 

Apathy and depressionare two distinct entities although it is not always easy to distinguish between those two symptoms. First, there is some overlap of symptoms: loss of interest, diminished pleasure in activities, or fatigue. Second, apathy could also be part of depression. On the other side, it has been suggested that apathy can occur in the absence of sadness, and depression can occur in the absence of apathy [41]. 

The relationship between the incidence of apathy and the decrease of dopamine has been suggested [42]. Dopamine is known to be implicated in motivational process, attributing an attracting character to things and stimulating willingness to act. Motivation and decision to act were found recovered after an increase of dopaminergic therapy dosage in apathetic PD patients [43, 44].

At the earliest stages of the disease, the occurrence of apathy is mostly dopamine dependant [45]. Whit more advanced stages of the disease the cognitive decline could contribute to the apathetic behaviour, through dysexecutive dysfunction (difficulties to plan an action, slowness of thinking). In this case, treatments acting on attentional capacities can be proposed. 

### 2.2. Dopamine Dysregulation Syndrome and Impulse Control Disorders

Also named hedonistic homeostatic dysregulation in PD, dopamine dysregulation syndrome (DDS) has been recently defined as compulsive use of dopaminergic drugs, associated with severe behavioral symptoms, and impaired social functioning [46, 47]. DDS consists of a craving or intense desire to obtain medication, even in absence of motor parkinsonian symptoms. 

Impulse control disorders (ICDs) are behavioral disorders characterized by failure to resist an impulse, inability to cut down and unsuccessful attempts to control a specific behavior. ICDs include pathological gambling, hypersexuality, compulsive shopping, and binge or compulsive eating. Another repetitive and compulsive behavior, the punding, has been described [48]. 

Pathologic gambling is a failure to resist the urge to gamble, with persistent and recurrent maladaptive gambling behavior despite deleterious consequences on familial, occupational, and social functioning. Hypersexuality is characterized by a preoccupation with sexual thoughts, frequent demands and desires, and Internet pornography use or contact with sex workers [49]. Compulsive shopping is characterized by maladaptive preoccupation of buying or shopping, whether impulses or behaviors that are experienced as irresistible, intrusive, and/or senseless, resulting in frequent buying of more than can be afforded [50]. Compulsive eating refers to an uncontrollable consumption of a larger amount of food than normal in excess of that necessary to alleviate hunger [51]. Punding consists on an intense fascination, with repetitive handling, examining, sorting, and arranging of objects [52].

ICDs occur in 15% to 20% of PD patients, and their prevalence has been found increased among patients treated with a dopamine agonist, compared with patients not taking a dopamine agonist [47]. More precisely, pathological gambling has been found in 5% of PD patients, compulsive sexual behaviour in 3.5%, compulsive buying in 5.7%, and binge-eating disorder in 4.3% [47]. Several ICDs can coexist in a given patient. Prevalence of DDS or punding behavior has not been studied as much as prevalence of ICD. About 4% of PD patients have been identified to have DDS [53]. Punding has been reported in 1.4% to 14% of PD patients according to different studies [48, 52]. 

In PD, ICDs appear in association with dopamine agonist therapy and are increased if combined with L-Dopa [54]. Nevertheless, individual vulnerability has been also evocated. The identified risk factors consist on a younger age, male gender, family history of addictive problems, depression, anxiety, and specific traits such as impulsivity and novelty seeking [55, 56].

Screening instrument can assist in determining if a clinically significant problem exists. However, international validated screening tools for all ICDs occurring with PD treatment are still under investigation [57, 58]. 

Several hypotheses have been proposed to underlie those abnormal behaviors. Neural substrates research highlighted an overactivity in brain areas critically involved in reward and reward-based learning, motivation, impulse control, decision making and memory processing, namely, the basal ganglia, the orbitofrontal cortex, the hippocampus, the amygdala, and the insula. The dopaminergic therapy may stimulate mesolimbic circuitry related to engagement in motivated behaviors seen in ICDs. In PD, ventral striatal dopamine is relatively preserved compared to dorsal striatal activity. Dopaminergic treatment titrated to alleviate motor dorsal striatal deficiencies may result in an “over-dosing” in ventral corticostriatal cognitive and limbic pathways. Indeed, hyperdopaminergic levels induced by overstimulation of relatively preserved mesocorticolimbic dopamine pathway could result in ICD, compulsive use of medication, and punding behaviors, in patients with individual predisposition [46, 55, 59]. Dopamine agonist is also known to enhance learning from rewarding outcomes and reinforce impulse choice. Impulse control disorders could be the result of dysfunction of an inhibitor pathway concerning assessment of negative consequences of an action [60, 61]. Thus, impulsive decisions associated with a distorted estimation of the consequences of actions (positive bias) could lead to abnormal behaviors with disastrous repercussions for everyday life.

Ethical issues and distress for subjects and their family (loss of employment and family break-up) have to be taken into consideration before initiating dopaminergic therapy in a young PD patient [62]. Many authors reported a dramatically improvement of the abnormal behavior after a dose reduction of the dopamine agonist therapy or a change to a different agonist [46, 63]. 

### 2.3. Cognitive Decline

It is important to distinguish mild cognitive impairment from dementia, the latter being present only in a moderate percentage of PD patients. In PD, very subtle cognitive disorders can develop insidiously within the first years of the disease. At this stage, cognitive deficits are not or few disabling in the context of daily life but, in a moderate proportion of cases, can progressively increase with the evolution of the disease. Cognitive disorders consist first of an intellectual slowing and difficulties to organize and manage the intellectual capacities, with preservation of global cognitive efficiency for long time [64]. Eventually, these disturbances can progressively increase with the time.

These cognitive disorders imply dysfunction of substantia nigra and nigrostriatal pathway. There is imbalance of striato-cortico-frontal pathways (which underlie the organisation of voluntary behaviors), causing a subcorticofrontal syndrome. Frontal subcortical syndrome in PD is implicated in all cognitive disorders, which are linked to dysfunction of the axis basal ganglia-frontal cortex. Frontal subcortical syndrome in PD is characterized by deficit of behavioral regulation in sorting or planning tasks, defective use of memory stores, and impaired manipulation of internal representation of visuospatial stimuli. There is a reduced speed of information treatment, psychomotor slowness, and also dysexecutive cognitive-behavioral syndrome.

Alteration of the ascending cholinergic and catecholaminergic neuronal systems may partly contribute to the frontal-lobe-like symptomatology associated with PD [64].

Dementia can be present at advanced stage of PD. Prevalence of dementia among PD patients is estimated between 20 and 30% according to the studies [65, 66]. In a specific study, dementia is associated with older age at the onset of the disease, male sex, greater severity of neurological symptoms, longer duration of disease, and greater disability [67]. Older age at the entry into the disease and severity of motor symptoms are significant predictors of dementia [67].

Prevalence of PD dementia in the general population aged over 65 years old is from 0.2% to 0.5% [65]. Estimation of the incidence of dementia in PD requires prospective population-based studies: a study conducted on a large cohort of nondemented patients with PD concluded that the risk for developing dementia is six times higher in patients with PD compared with the general population [68].

Cortical activity of choline acetyltransferase, a marker of cortical cholinergic activity, is reduced in PD [69]. Cholinergic inhibitors have been shown to have a positive effect on cognition in PD dementia [70]. 

### 2.4. Hallucinations, Delusions, and “Psychosis” 

Psychotic symptoms (hallucinations, loss of insight, and delusions) affect most patients with PD. Psychosis is defined as hallucinations, delusions, or both, in patients with clear sensorium [71]. In PD, incidence of hallucinations is 80 for 1000 for year [72], more frequent in aging patients.

Hallucinations occur usually in a normal state of consciousness, without delirium, and have a chronic course [73]. The prevalence of complex visual hallucinations ranges from 22 to 38% [74]. Risk factors for hallucinations are older age, long duration of the disease, cognitive impairment, severity of PD symptoms, sleep disorders (somnolence), and visual disorders [74]. Risk of psychotic symptoms is increased in late onset PD, in patients taking high doses of dopaminergic drugs and suffering of REM sleep behaviour disorder (RBD). Hallucinations must be identified by systematically questioning the patient.

Visual hallucinations are surprising, but their intensity is quite variable. Benign hallucinations are limited to presence sensation, passing lights or visions at periphery of the visual field, with great tolerance by the patient. Conversely, elaborated hallucinations are usually disabling because their disturbing nature (wild animals, fantastic human creatures). Illusions consist of transformation of a real image and occur more often in the dark. Auditive, olfactive, or tactile hallucinations are less frequent [73]. Visual perception disorders and poor visual discrimination are often associated with hallucinations [75]. In 10 parkinsonian patients with severe hallucinations, hallucinations occurred with the irruption of periods of REM sleep during the day [76].

Hallucinations may occur in the presence of delusions with delirious convictions and can cause significant distress to the patients and their surroundings. Hallucinations are not recognized by the patient when alterations of cognitive faculties or dementia are also present. This cognitive decline plays a role in chronic evolution of hallucinations. Hallucination episode can even lead to the institutionalisation of an aged patient. Hallucinations are a risk factor for the development of cognitive impairment and dementia [77]. This association with increased disability and dementia place PD psychosis within symptoms signalling a serious disease course [72, 78].

Despite hallucinations can be present in drug-naïve patients, they are mostly induced by dopaminergic therapies, especially dopamine agonists, but there is no simple dose-effect relationship between dopaminergic therapy and hallucinations [79]. Improvement of hallucinations follows the decrease of dopaminergic treatment. 

In addition to the iatrogenic hypothesis, it is now admitted that specific factors linked to the disease may contribute to hallucinations. Hallucinations could be related to hypersensitization of dopamine receptors in frontal and limbic areas. Studies with functional MRI (fMRI) have confirmed the role of visual associative cortical areas, with dysfunction of networks between these areas and frontal lobes, on one hand, and subcortical areas, one the other hand [80]. Lesions in the pedunculopontine nucleus, locus cœruleus, dopaminergic raphe nucleus, lingual gyrus, and superior parietal lobe are also implicated in the pathophysiology of hallucinations [81]. Moreover, accumulation of Lewy bodies in amygdala, parahippocampal cortex, and inferior parietal cortex is described in case of hallucinations [82]. 

Treatment of hallucinations often necessitates reducing the dopaminergic treatment, despite the risk of recrudescence of the motor parkinsonian symptoms (increase of akinesia). Most of the time, the treatment implies a combination of strategies addressing the possible contributing factors in individual cases (infection, metabolic disturbances, etc.). All pharmacological treatment must be revised and adjusted if they are believed to contribute to the symptomatology, especially the antidepressants, anxiolytic, anticholinergic, and benzodiazepines. Small doses of atypical antipsychotics may be used. In practice, clozapine remains the most recommended treatment [83].

### 2.5. Pain

Pain is the more frequent NMS at onset of the disease, and its prevalence increases with the duration of disease [84, 85]. Pain may precede the onset of motor parkinsonian symptoms. The prevalence of all types of pain is high, but variably assessed. In a recent case-control study [86], the frequency of pain was greater in PD patients (69.9%) than in controls (62.8%). After adjustment concerning osteo-articular comorbidity, pain is estimated to be twice more frequent in PD patients than in patients without PD [87]. Chronic pain is present in 30% to 85% of patients [87]. Pain is directly attributed to PD in 46% of patients [88].

Clinical features of pain, prevalence, pathophysiology, and management of pain are well summarized in a review published by Ha and Jankovic, in 2011 (Table 2) [89]. A common type of pain is musculoskeletal pain, resulting from a combination of factors such as rigidity, arthralgic pain, skeletal deformity, and mechanical factors [90]. Dystonic and nondystonic pains are both considered nonmotor features in PD [86]. Dystonic pain is reported in 40% of patients [91] and is dopa responsive. Therefore, painful spasms typically involving feet (such as “off” medication dystonia, or early morning dystonia) are improved by adaptation of levodopatherapy. In PD, nondystonic types of pain include arthralgic, cramping, peripheral neuropathic pain, and central neuropathic pain (burning, tingling, formication, or bizarre quality). Radicular or neuropathic pains are less frequent. Central neuropathic pain is poorly localized, constant, boring, ineffable, and not limited to a dermatome or nerve distribution [91]. Pain is a troublesome symptom, but often not recognized and not adequately treated. 

Pathophysiology of pain involves the corticobasal ganglia-thalamic circuits for multisensory integration of pain. Dopaminergic nigrostriatal lesion leads to modification of pain perception. In PD, central pain modulation process is altered, with predominant hyper activation of nociceptive median circuit (affective component of pain). Nociceptive threshold is decreased in PD [92] but is in partly normalized by levodopa. In fact, levodopa changes the pain thresholds [93]. 

Treatment of pain depends on the type of pain and usually requires a multidisciplinary approach. Not only levodopa-related dystonia, but also other types of pain (musculoskeletal pain related to rigidity and akinesia, akathisia), respond to optimization of levodopatherapy. Pain responds frequently to dopaminergic medication also; reduction of pulsatile dopamine receptors stimulation improves pain like levodopa-related dystonia, but also musculoskeletal pain. Botulinum toxin is used for treatment of focal painful dystonia [90]. Other neurotransmitters may also contribute to pain in PD, such as noradrenalin, serotonin, glutamate transmission and opioïd systems [94]. The benefit of doluxetine (inhibitor of noradrenalin and serotonin) on primary pain symptoms in PD is an example of this involvement of other neurotransmitters [95].

### 2.6. Sleep Disorders

Sleep disorders are present in about 66% to 90% of PD patients. Sleep disorders are more frequent in subjects suffering from advanced disease. In PD, sleep disorders are multiple and have varied substratum. RBD is a substantial risk factor for development of PD and occurs in one third of patients with PD. 

Nocturnal disturbances can be defined by three types as follows.

- PD-related motor symptoms, including nocturnal akinesia, early morning dystonia, painful cramps, tremor, and difficulty turning in bed. The optimization of dopatherapy and use of long acting agonist is useful.
- Nocturnal behavioural disturbances (agitation, confusion) related to the presence and/or the treatment of PD psychiatric symptoms (hallucinations, depression, and anxiety). Adjustment of the drugs that may induce these disturbances, treatment of depression and anxiety, prevention of confusion in patient with cognitive impairment and specific treatments should be used for each patient, according to the type of sleep disorder.
- Other sleep disorders: insomnia (fragmentation of sleep) (37%), RBD (30%), restless legs syndrome (RLS) (15%), periodic movements of limbs during sleep (PMLS), and excessive day time sleepiness (EDS) (21%) [96].

Insomnia consists of difficulty falling asleep, with fragmentation of sleep and frequent awakenings and early awakening in the morning. The prevalence of insomnia in PD patients is from 40% to 80%. Motor disability plays a major role, particularly persistence of motor PD symptoms, akinesia (difficulties to turn in bed), rigidity, muscular contractures, dystonia (early morning dystonia), and pain linked to “off” periods. Others reasons for insomnia are nocturnal urinary disturbances (nocturia), RLS, and respiratory disorders. Effectively, 20% of PD patients are suffering from obstructive sleep apnea syndrome or SAS [97]. Excluding aging, main risk factors for insomnia are presence of depression or anxiety, duration of levodopatherapy, and duration of the disease. 

In PD, abnormal movements during sleep can be single (such as myoclonia) or complex (such as RLS and PMLS). They can lead to frequent awakenings. PMLS are rhythmic movements with extension of big toe or flexion of ankle and knee. They are present in 15% of cases and are improved by levodopa. RLS (20% of PD patients) consists in a disabling sensation, deeply in legs, with urge to move, and with transitory decrease of these discomfort after moving [98]. RLS explains falling asleep difficulties and is often associated with PMLS. RLS is improved by slight dose of dopaminergic treatment in the evening. Nocturnal akathisia is associated with sensation of impatience, with difficulty to stay motionless. 

RBD (from 25 to 50% of patients) is associated with loss of atonia (normal muscle tonus suppression) during REM sleep, with direct access to dream content and with acting out of the dream while asleep. A motor and verbal expression of dreams, with violent dream-enacting behaviors, happens in the absence of motor PD symptoms. Hence, a complex body language appears, pointed to a goal, nonstereotyped, with rapid and saccadic movements and risk of injuries or falls. RBD is equivalent to an active dream. One third to half of PD patients present RBD, often in association with hallucinations in a given patient. In PD, the presence of RBD is a risk factor for hallucinations and dementia. RBD is present in one-half of cases, before the onset of motor symptoms of PD. The presence of RBD is predictive factor of PD; from 20 to 40% of patents develop neurodegenerative disease (synucleinopathy) within a mean 5 years [99, 100].

Parasomnias are undesirable behaviours occurring exclusively during sleep or exaggerated by sleep. Parasomnias include vivid dreams, altered dream content, nightmares, night terrors, nocturnal vocalizations, RBD, nocturnal hallucinations, sleep talking, somnambulism, and panic attacks [101]. The most relevant parasomnias in PD are RBD and nocturnal hallucinations. Most parasomnias are few disabling, and the patient even does not know about it. This is not true for the bed partner, who witnesses these nocturnal disturbing behaviors and, then, constitutes a great informative source for the physicians. These phenomenona, particularly vivid dreams and hallucinations, can be amplified by levodopa. The clinician should pay attention to the occurrence of vivid dreams because it is the forewarning sign of hallucinations and levodopa-induced “psychosis.”

Excessive daytime somnolence (EDS) is defined as a symptomatic daytime somnolence with frequent sleep periods. The causal factors of EDS areinsomnia, depression, dysautonomia (nocturnal hyperhydrosis and nocturia), and some drug therapy. The frequency of EDS and sleep attacks is variably assessed according to studies, from 20% to 50% of patients [102]. EDS appears more frequently in advanced PD, associated with cognitive disorders or depression, long duration of levodopa therapy and hallucinations. Indeed, somnolence is at increased risk during levodopatherapy in vulnerable subjects such as aging patients with cognitive decline. EDS is linked to individual susceptibility in PD.

Abrupt sleep access, named “sleep attacks,” consists in abruptly transition between awakening and sleep state without prodrome. “Narcolepsy without cataplexy” is not infrequent in PD patient [76]; hallucinations coincide with access of diurnal REM sleep in PD patients presenting hallucinations and narcolepsy-like sleep disorder [99].

Dopamine agonists seem to increase the risk of EDS and “sleep attacks.” The clinicians must pay attention to the possibility of sleep attacks because the consequences can be very serious if they occur during driving.

The effects of dopaminergic treatment on insomnia are variable. Mostly, improvement of sleep follows an increase of levodopa dosage. However, too high or too late intake of dopaminergic therapy can promote insomnia. Sustained dopaminergic stimulation by the use of slow-release levodopa or long-acting dopaminergic agonists improves nocturnal akinesia and insomnia [103]. RBD can be treated by low dose of clonazepam. RLS is improved by low dosage of dopamine agonist, or opioids (codeine), or anticonvulsants (clonazepam) [104]. 

### 2.7. Dysautonomia

Autonomic symptoms in PD include orthostatic hypotension, constipation, bladder and sexual dysfunction as well as sweating abnormalities [105]. Autonomic failure may be an early feature of PD although it is more typically associated with advanced stage of the disease. Prevalence of autonomic disturbances is variably assessed, ranging between 14% and 80%, and these symptoms are better evaluated with the SCOPA-AUT scale [106].

#### 2.7.1. Genitourinary Dysfunction

Lower urinary tract symptoms, such as urinary frequency, nocturia, and urinary incontinence, are frequent in PD, interesting one-half of patients, thereby affecting the quality of life. Urinary symptoms can appear early in the disease [8]. These symptoms are in relation to detrusor hyperactivity caused by an altered dopamine basal ganglia circuit, which normally inhibits the micturition reflex. Lower urinary tract symptoms increase with motor disability, possibly in relation to dopaminergic denervation. Anticholinergic agents for treatment of the detrusor hyperactivity must be cautiously used in PD [107].

Sexual dysfunction in PD is frequently reported, in one-half to two third of patients [108]. All the following aspects of sexual function can be altered: decrease of libido, decrease of sexual intercourse, decrease of orgasm, decrease of erection, and ejaculation. Hypothalamic dysfunction is mostly responsible for sexual dysfunction, in PD, via dopamine-oxytocin pathways, normally promoting libido and erection [107]. Among men with erectile at baseline, a retrospective study concludes to a 3.8-fold increase in risk of developing PD [109].

#### 2.7.2. Orthostatic Hypotension in PD

Orthostatic hypotension is defined as a fall of at least 20 mm Hg fall in systolic pressure and/or 10 mm Hg fall in diastolic pressure within three minutes of standing [110]. Blood pressure should be measured after 15 minutes of supine rest, and thereafter every minute for five minutes while standing, with measurement of the heart rate [111]. There is an orthostatic hypotension in 20%–58% of PD patients [112]. PD is a cause of primary autonomic failure with presence of peripheral postganglionic sympathetic dysfunction, which can be demonstrated by MIBG cardiac scintigraphy [113]. Early onset of symptomatic orthostatic hypotension is considered as exclusion criteria for idiopathic PD [114]. Symptoms of autonomic dysfunction correlate with disease duration and severity. Additional risk factors are male sex, increased age, and large doses of levodopa [115]. Antiparkinsonian medications, such as levodopa, dopamine agonists, and I-MAO B, can induce orthostatic hypotension, independently of the disease.

Treatment of orthostatic hypotension related to PD consists systematically of nonpharmacological strategies (gradual stand-up, sleeping with head-up position, fragmentation of meals, avoidance of high carbohydrates or low sodium diet, large quantities of fluid intake, elastic stockings). Drugs acting on postsynaptic adrenoreceptors, inhibiting vasodilating factors, and acting on volemia have not been evaluated in PD and should be used only with respect of contraindications (supine arterial hypertension) and cardiovascular status [111].

#### 2.7.3. Constipation

Constipation is more frequent in patients who subsequently develop PD than in control [116]. Precession of motor symptoms by constipation is demonstrated [117]. Disabling constipation (from 45% to 60% of PD patients) predominates on the later stage of disease, with difficulty in defecation and reduced number of bowel movements, in comparison with early stages [118].

Treatment of constipation is identical to other causes of constipation (increase dietary fibbers, avoid dehydration, increase exercise, and laxatives), but also requires adaptation of levodopatherapy in patients with motor fluctuations. 

## 3. Nonmotor Fluctuations (NMFs)

NMFs are often underappreciated [119]. Motor fluctuations, in relation with decrease of dopamine striatal transmission, are characterized by the reemergence of parkinsonian motor symptoms during “off” state, such as the “wearing off” or the “on-off” phenomenon. NMSs occur in the “off” periods, as well as during “on” state. NMFs vary according to plasma dopaminergic concentrations, such as motor fluctuations. In fact, dopaminergic systems are involved, but other neurotransmitters could be interested, such as serotonin or noradrenalin, via modulation by dopaminergic pathways [120]. 

NMFs are sometimes predominant (example, anxiety). They consist of autonomic disorders (blood pressure changes, urinary frequency and urgency, thermoregulation disorders), cognitive/psychiatric (anxiety, depression, and slowness of thinking), and sensory complaints (pain, fatigue, and akathisia) [5, 121] (Table 3).

In a study questioning 50 PD patients, Witjas and col conclude that all patients have at least one type of NMFs, and that more than one quarter are more disabling with NMFs than with motor fluctuations [5]. The most frequent NMF is anxiety (66%), then drenching sweats (64%), slowness of thinking (58%), fatigue (56%), akathisia (54%), irritability (52%), and hallucinations (49%). Autonomic symptoms consist of excessive sweating, swallowing difficulties, abdominal discomfort, urgency, or urinary frequency, in “off” periods. 

### 3.1. Diagnosis Tools for NMS

NMSs are frequently not accurately assessed in everyday clinical practice. The routine administration of screening tools, followed by a further detailed evaluation, could notably enhance the NMS identification.

Currently, the following instruments have been developed and are helpful in clinical practice.

(a) The NMSQuest (NMSQ), a self-administered screening tool, consists of 30 items, classified in 10 domains [9, 123, 124]. These domains are cardiovascular, sleep/fatigue, mood/cognition, perceptual problems/hallucinations, attention/memory, gastrointestinal, urinary, sexual, and miscellaneous. The frequency of NMS, by reporting these with NMSQ, was assessed with clinical scales of specific NMS. The impact of NMS on quality of life was measured. In multivariate analysis, depression has the stronger association with health-related quality of life (Hr-QoL), followed by fatigue, thermoregulatory, gastrointestinal, and cardiovascular autonomic function (orthostatic hypotension), daytime somnolence, and urinary problems.

Autonomic dysfunction, psychiatric complications, pain, fatigue, and sleep problems are major correlates of poor Hr-QoL [125].

(b) The non-motor symptoms scale (NMSS), consisting in 30 items instrument, is rated by clinicians, to explore 9 domains (cardiovascular, sleep/fatigue, mood/apathy, perceptual problems/hallucinations, attention/memory, gastrointestinal, urinary, sexual dysfunction, and miscellaneous) [126]. Items are scored for frequency and severity. NMSS is specifically designed for the comprehensive assessment of NMS in patients with PD. Psychometric attributes of NMSS were confirmed by the study of Martinez-Martin et al. in 2009 [124]. There is a close association between NMSS and PDQ 39 [124], and a correlation between NMSS and NMSQ and PDQ8 [126]. 

(c) The scales for outcomes in PD programmes (SCOPA) to address the unmet need of assessment tools for NMS in PD are validated for motor and nonmotor domains as follows: scale for outcome of PD-cognitive scale (SCOPA-COG) [127], scale for outcome of PD-sleep scale (SCOPA-Seep scale), and scale for outcome of PD-autonomic scale (SCOPA-AUT) [128, 129]. An available patient reported daily diary for measurement of both fluctuating motor and non-motor PD symptoms which have been identified and validated: it consists in the “Revised SCOPA-Diary Card (Scales for outcomes of PD-Diary-Card”) [130].

(d) The following rating scales have been recommended by the Movement Disorder Society's Task Force on Rating Scales in Movement Disorders: 

(i) Apathy Scale (AS);

(ii) Beck Depression Inventory (BDI); Geriatric Depression Scale (GDS), Hamilton Rating Scale for Depression (HAM-D), Hospital Anxiety and Depression Scale (HADS); 

(iii) Montgomery Asberg Depression Rating Scale (MADRS); 

(iv) SCOPA-AUT for assessment of autonomic disorders;

(v) Parkinson's disease Quality of Life Scale (PDQUALIF)

(vi) Movement Disorder Society-Unified Parkinson's Disease Rating Scale (MDS-UPDRS): the Movement Disorders Society's revision of the UPDRS (MDS-UPDRS) is an improved scale, taking in count NMS symptoms. MDS-UPDRS includes mainly non motor domains [131]. Validation of the MDS-UPDRS Part 1 for non motor symptoms in Parkinson's disease has demonstrated a strong relationship with a composite score of validated scales for NMS aspects of PD [132].

(vii) Brief Psychiatric Rating Scale (BPRS); Neuropsychiatric Inventory (NPI); Positive and Negative Syndrome Scale for Schizophrenia (PANSS); Scale for the Assessment of Positive Symptoms (SAPS); and Scale for Assessment of Negative Symptoms (SANS).

(e) Nonmotor fluctuation assessment instrument (NoMoFA) is an instrument of assessment of on non motor fluctuations in PD [133]. NoMoFA is proposed as a patient-based instrument for detection of NMFs [133].

(f) A task force that has been commissioned by the Movement Disorders Society [134] recommended six scales for sleep assessment in PD: (a) the PD sleep scale (PDSS) and the Pittsburg sleep quality index (PSQI) for rating overall sleep problems; (b) the SCOPA-sleep (SCOPA) for rating overall sleep problems both to screen and to measure severity, and daytime sleepiness; (c) the Epworth sleepiness scale (ESS) for rating daytime sleepiness; (d) the inappropriate sleep composite score (ISCS), for sleepiness or sleep attacks; (e) the Stanford sleepiness scale (SSS; non validated) for rating sleepiness and measure severity at specific moment.

(g) Questionnaire for impulsive compulsive disorders (QUIP) is validated [135].

## 4. Premotor NMS

Some NMS can be the earliest symptoms of PD [136, 137], occuring in the premotor phase, that is, years before the development of akinesia, rigidity, or tremor. The identification of premotor NMS opens up the possibility of presymptomatic diagnosis of PD. Premotor NMS are olfactory dysfunction, dysautonomia, mood disorders, and sleep disorders (Table 4).

Hyposmia or olfactory loss is a disorder of odour detection, identification, and/or discrimination, probably due to the alteration of olfactory bulb, anterior olfactory nerve, amygdala and perirhinal cortex. More than 90% of PD patients present hyposmia or anosmia. Association between impaired olfaction and future PD is proved. Olfactory dysfunction may predict and can be considered as an early marker of PD; patients affected by hyposmia present a 10% increased risk of developing PD within 2 years of the onset of olfactory dysfunction, compared to their asymptomatic relatives [138, 139]. Olfactory loss can even predate the development of clinical PD by up to 4 years. Whatever hyposmia precedes or occurs simultaneously with neuronal loss of the substantia nigra requires clarification [139]. 

Dysautonomia can also precede motor symptoms. In fact, constipation appears to be common in PD patients at onset of the disease and can antedate the development of parkinsonian symptoms by more than 10 to 18 years [117]. A recent study from the Mayo Clinic found an odds ratio of 2.48 for the presence of constipation preceding PD, with a lag time between the onset of constipation and motor symptoms of 7.9 years [140]. Constipation could be related to the underlying pathological process of PD, such as the deposition of alpha synuclein at the dorsal nucleus of the vagus and at the enteric plexus [141, 142]. 

Depression can predate PD diagnosis by 3 to 6 years before diagnosis [143]. It is estimated that depressed patients have a 2.2- to 3.2-fold higher risk for developing PD than nondepressed subjects [30], but depression is not predictive of future PD development.

Sleep disturbances are from 1.5 to 3.5 times more common in established PD than in healthy controls or patients with other chronic diseases [144, 145]. Subjects with idiopathic RBD are at high risk of developing PD. In a study following 40 patients with idiopathic RBD, 19% to 38% have developed PD after 5-year followup [146]. In about 20% of PD patients with RBD, the reported onset of RBD antedates that of Parkinsonism (PD and parkinsonian syndromes) by several years [147]. About 20% of patients with idiopathic RBD, characterized by loss of normal atonia with REM sleep, will develop PD or dementia within 10 years [148]. Locus coeruleus and pedunculopontine nuclei lesions seem to be responsible for RBD in PD.

Relation between premotor symptoms and pathological process in PD is actually discussed. The theory of Braak staging [149] could explain extranigral early lesions. Alpha-synuclein pathology begins in the anterior olfactory nucleus and the lower brain stem (dorsal motor nucleus of the vagus) and spreads upward from the medulla, only affecting the substantia nigra compacta at Braak stage 3, and then purses an ascending course up to cortical areas. The pathology also affects the peripheral autonomic nervous system involving sympathetic ganglia, cardiac sympathetic afferents, and enteric nervous system [150].

## 5. Conclusion

With the NMSQuest, the prevalence of NMS is high. NMS are present across all disease stages and increase with duration and severity of the disease [123]. A study of prevalence of NMS in more than 1000 patients, using a semistructured interview, reveals that all patients complained of a range of NMS, across all stages of the disease [96]. 

 NMS are underestimated in contrast to motor symptoms. A range of NMSs are often undeclared, and treatments are inappropriate [151]. Depression, anxiety, and fatigue are not identified in one half of patients, sleep disorders in more than 40% [152]. Then, the importance to detect NMS appears because they are less known than motor disturbances, and not so well treated.

Indeed, during routine consultation, two errors must be avoided: (a) to take into account the clinical presentation at the moment of examination and not consider the condition of the patient in daily life; (b) to take into account only anamnesis and appearance of motor symptoms, and forget to look for NMS. The patient itself can be embarrassed to declare some NMS, such as ICDs or hallucinations.

Nonmotor symptoms are determinant for the quality of life of patients. The impact of NMS on quality of life can be evaluated with the aim of the NMSS: the most frequent complains are being nocturia (68%), fatigue (66%), and dribbling saliva (57%) [153]. Severity of the disease (Hoehn and Yahr stade >3) is associated with reduction of quality of life, increased disability, and increased NMS prevalence [154]. Depression has leave the impact of motor signs on quality of life. Anxiety and other non motor symptoms are also important separate determinants of poor health status in PD [155]. PD patients with cognitive dysfunction are at risk for deterioration of quality of life over time [156]. At advanced stage of the disease, the treatment of NMS should improve the quality of life [157].

## Figures and Tables

**Table 1:** Major nonmotor symptoms (NMS) in PD (adapted from [3]).

(a) Neuropsychiatric symptoms:	
(1) Depression	
(2) Anxiety	
(3) Apathy	
(4) Hallucinations, delusions, illusions	
(5) Delirium (may be drug induced)	
(6) Cognitive impairment (dementia, MCI)	
(7) Dopaminergic dysregulation syndrome (usually related to	
levodopa)	
(8) Impulse control disorders (related to dopaminergic drugs).	
(b) Sleep disorders:	
(1) REM sleep behaviour disorder (possible premotor	
symptoms)	
(2) excessive daytime somnolence, narcolepsy type “sleep	
attack”	
(3) restless legs syndrome, periodic leg movements	
(4) insomnia	
(5) sleep disordered breathing	
(6) non-REM parasomnias (confusional wandering)	
(c) Fatigue:	
(1) central fatigue (may be related to dysautonomia)	
(2) peripheral fatigue.	
(d) Sensory symptoms:	
(1) pain	
(2) olfactory disturbance	
(3) hyposmia	
(4) functional anosmia	
(5) visual disturbance (blurred vision, diplopia; impaired	
contrast-sensitivity).	
(e) Autonomic dysfunction:	
(1) bladder dysfunction (urgency, frequency, nocturia)	
(2) sexual dysfunction (may be drug-induced)	
(3) sweating abnormalities (hyperhydrosis)	
(4) orthostatic hypotension.	
(f) Gastrointestinal symptoms:	
(1) dribbling of saliva	
(2) dysphagia	
(3) agueusia	
(4) constipation	
(5) nausea	
(6) vomiting.	
(g) Dopaminergic drug-induced behaviour NMS:	
(1) hallucinations, psychosis, delusions	
(2) dopamine dysregulation syndrome	
(3) impulse control disorders.	
(h) Dopaminergic drug-induced other NMS:	
(1) ankle swelling	
(2) dyspnea	
(3) skin reactions	
(4) subcutaneous nodules	
(5) erythematous	
(i) Nonmotor fluctuations:	
(1) dysautonomia	
(2) cognitive/psychiatric	
(3) sensory/pain	
(4) visual blurring	
(j) Other symptoms:	
(1) weight loss	
(2) weight gain.	

**Table 2:** Classification of pain in PD adapted by [89].

Musculoskeletal: aching, cramping, arthralgic, myalgic sensations in joints, and muscles; may exacerbated by parkinsonian rigidity, stiffness, and immobility, postural abnormalities, and relieved by mobility; may be associated rheumatologic and orthopaedic disease. May fluctuate with medication dosing, and improve with levodopa.	
Dystonic: associated with sustained twisting movements and postures; muscular contractions often very forceful and painful; may fluctuate closely with medication dosing: wearing off dystonia, early morning dystonia, peak-dose dystonia, diphasic dystonia.	
Radicular/Neuropathic: pain in a root or nerve territory, associated with motor or sensory signs of nerve or root entrapment.	
Central or primary pain: burning, tingling, formication, “neuropathic” sensations, often relentless and bizarre in quality, not confined to root or nerve territory; pain may have an autonomic character, with visceral sensations or dyspnea, and vary in parallel with the medication cycle as a non-motor fluctuation; not explained by rigidity, dystonia, musculoskeletal or internal lesion.	
Akathisia: subjective sense of restlessness, often accompanied by urge to move; may fluctuate with medication effect, and improve with levodopa.	
Others types of pain: oral and genital pain; burning mouth or vagina syndrome; may represent a sensory wearing off and may improve with L-dopa.	

**Table 3:** Nonmotor fluctuations in Parkinson's disease (modified from [121, 122].

(1) Neuropsychiatric	
(a) Mood: anxiety, depression, panic attacks, apathy,	
moaning/screaming, and fatigue	
(b) Psychotic symptoms: visual hallucinations, delusions,	
paranoia, hypomania/mania, dopamine dysregulation	
syndrome, euphoria, and agitation	
(c) Cognitive dysfunction	
(2) Autonomic	
(a) Thermoregulation: sweating, facial flushing, pallor,	
hyperthermia	
(b) Dysphagia, dribbling of saliva, dry mouth, belching,	
nausea, abdominal bloating discomfort, constipation, and	
anismus	
(c) Urinary frequency and urgency	
(d) Blood pressure changes, tachycardia	
(e) Dyspnea, cough, stridor	
(f) Peripheral oedema	
(g) Hunger	
(h) Pupillary dilatation	
(3) Sensory	
(a) Pain	
(b) Internal tremor	
(c) Akathisia, restless legs syndrome	
(d) Sensory dyspnea	
(e) Numbness, dysesthesia	

**Table 4:** Premotor symptoms (adapted from [136]).

Strongest evidence:
(i) Olfactory deficit
(ii) Constipation
(iii) Sleep disorders (EDS, RBD)
(iv) Depression
Suggested links:
(i) Other autonomic dysfunction (cardiac…)
(ii) Anxiety
(iii) Visual disturbances
(iv) Cognitive changes
(v) Restless legs syndrome
(vi) Apathy
(vii) Fatigue
(viii) Personality traits
